# Supplementary material for: Vibrio type III secretion system 2 is not restricted to the Vibrionaceae and encodes differentially distributed repertoires of effector proteins
Source: Microb Genom. 2023 Apr 5;9(4):mgen000973. doi: 10.1099/mgen.0.000973 (PMC10210961; doi:10.1099/mgen.0.000973)
Supplement: Supplementary material 1 [file mgen-9-973-s001.pdf]

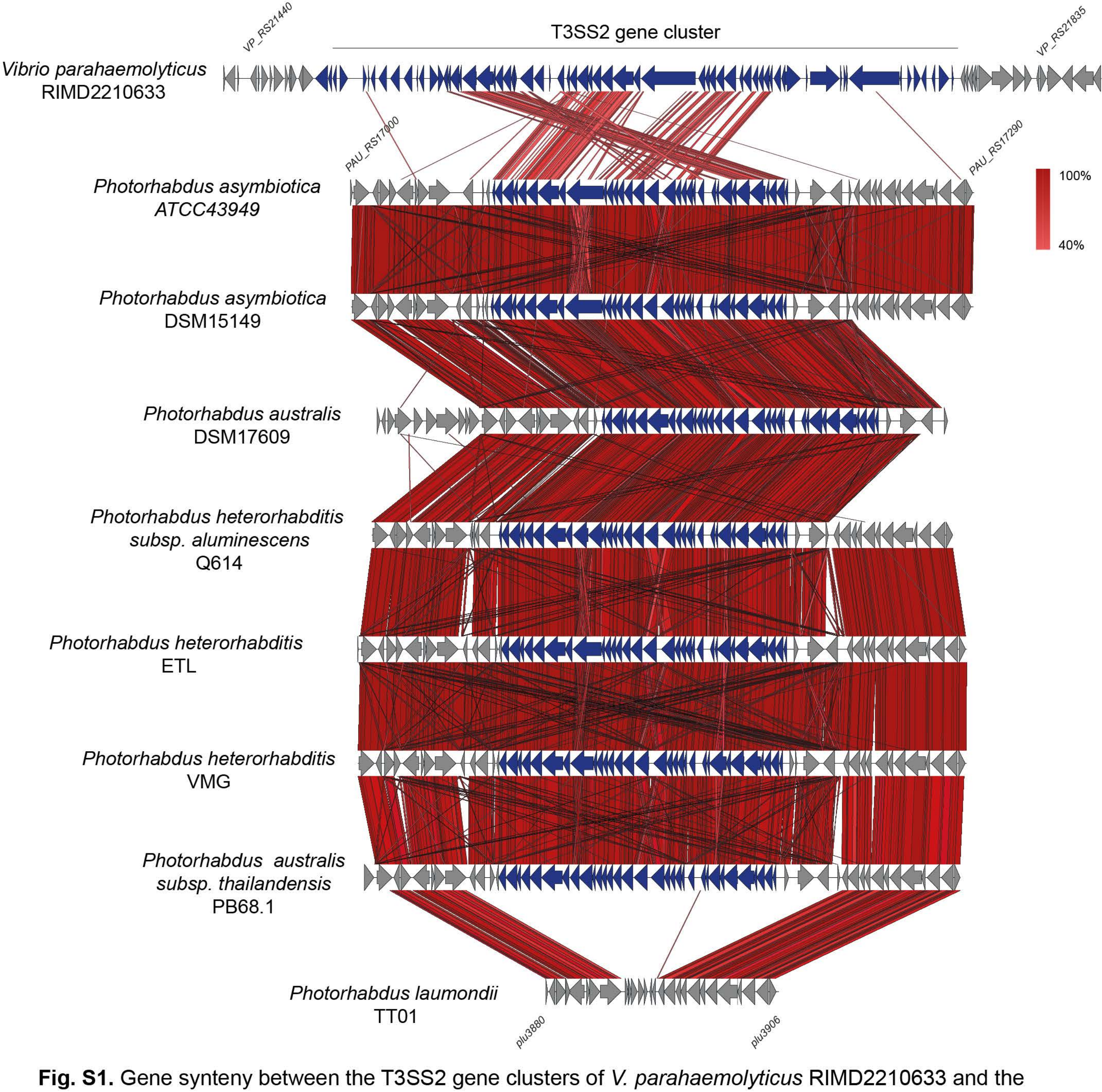

Fig. S1. Gene synteny between the T3SS2 gene clusters of *V. parahaemolyticus* RIMD2210633 and the identified T3SS2 gene clusters of Photorhabdus species. tBLASTx alignments were performed and visualized using Easyfig. Genes encoding T3SS-related genes are highlighted in blue.

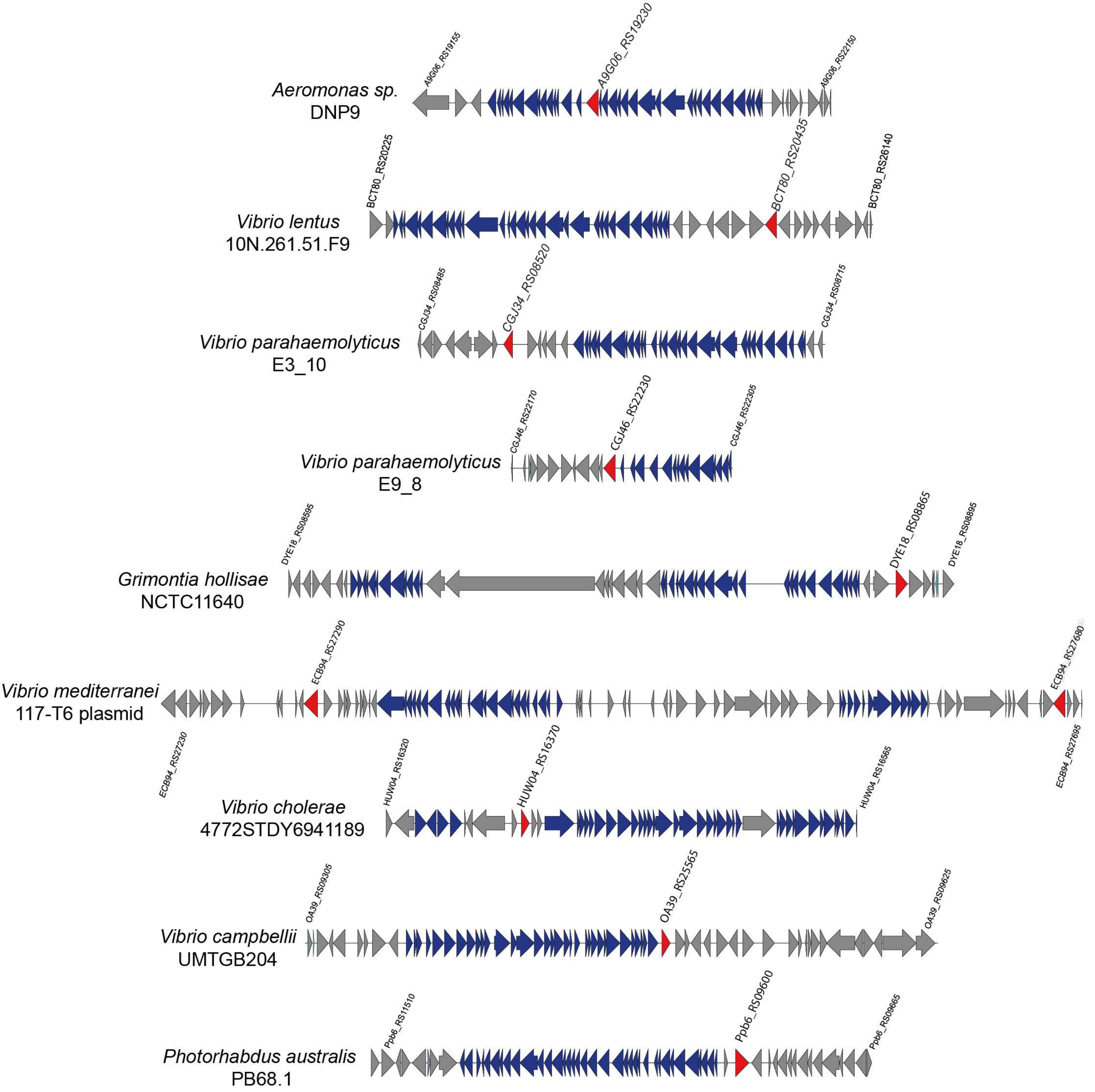

Fig. S2. Genetic context of novel effector candidates in representative T3SS2 gene clusters. The T3SS2 gene clusters are shown in blue, and each novel effector candidate is highlighted in red.

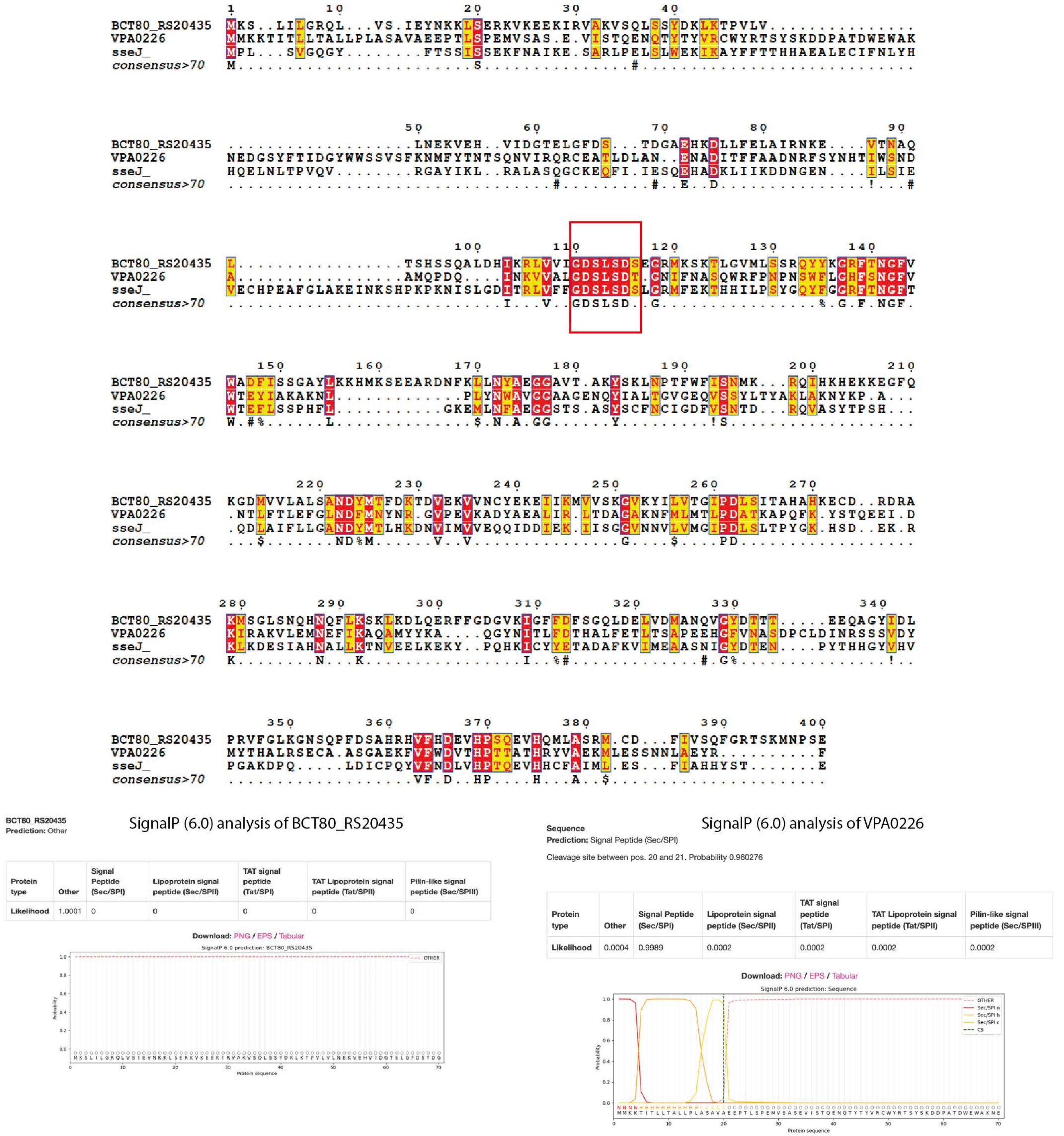

Fig. S3. Multiple sequence alignment of the amino acid sequence of the predicted effector BCT80_RS20435 and the secreted lipase VPA0226 and the Salmonella T3SS effector protein SseJ. The alignment was performed using T-Coffee Expresso and visualized by ESPript 3.0. Amino acids within a red background correspond to positions with 100% identity; amino acids with a yellow background correspond to positions with >70% identity SignalP analysis shows that BCT80_RS20435, unlike VPA0226, does not harbor Sec or TAT secretion signals.

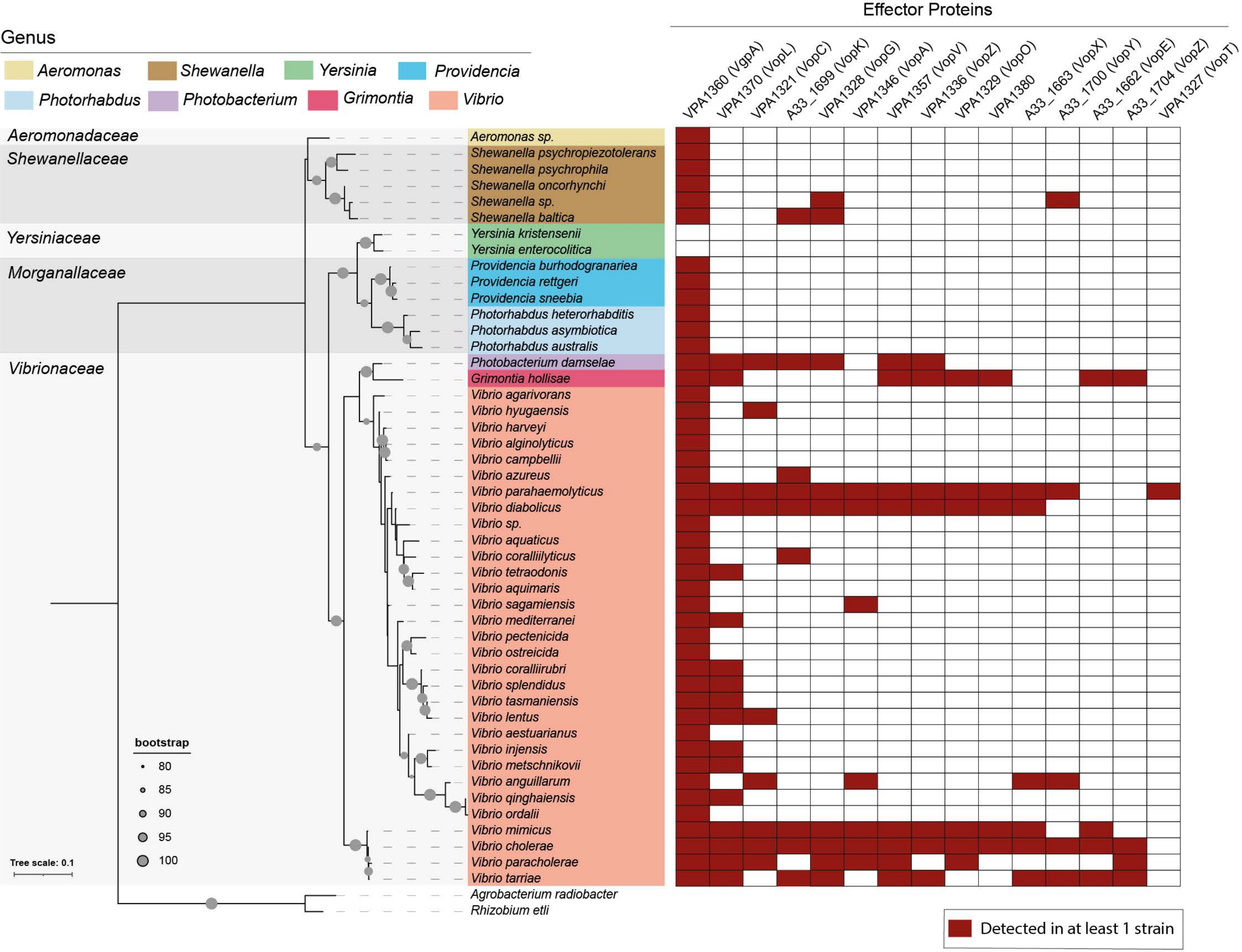

Fig. S4. Distribution of T3SS2 effector proteins among bacterial species. The distribution matrix shows the detection of each effector protein in at least one bacterial strain in red. The phylogenetic tree is the same tree as Fig 2.
